# Robotic repair of a traumatic Spigelian hernia

**DOI:** 10.1093/jscr/rjaf515

**Published:** 2025-07-15

**Authors:** Benson Li, Brandon Larson, Laura Crankshaw

**Affiliations:** Department of Surgery, Summa Health System, 141 N Forge St, Akron, OH 44304, United States; Department of Surgery, Summa Health System, 141 N Forge St, Akron, OH 44304, United States; Department of Surgery, Summa Health System, 141 N Forge St, Akron, OH 44304, United States

**Keywords:** Spigelian hernia, blunt, trauma, robotic repair

## Abstract

Spigelian hernias are a rare type of hernia that can be either congenital or acquired from weakening of the semilunaris line. Spigelian hernias are often difficult to diagnose due to the lack of an obvious defect on physical exam. This report presents the case of a traumatic Spigelian hernia in a 59-year-old after a fall from a tree onto a fence. Computed tomography imaging demonstrated several rib fractures as well as an 8 cm fat-containing Spigelian hernia. Given the risk of bowel herniation, this was repaired during the same admission via a robot-assisted approach with primary repair of the defect and mesh placement in an intraperitoneal onlay mesh fashion. At the patient’s postoperative visits at 1, 3, and 8 months, there was no evidence of hernia recurrence. This case highlights the robotic intraperitoneal onlay mesh technique as a safe and durable option for the repair of traumatic Spigelian hernias.

## Introduction

Spigelian hernias are exceedingly rare, accounting for only 0.12%–2% of all abdominal hernias. The Spigelian fascia is defined by rectus abdominis aponeurosis medially and the semilunar line laterally. Risk factors for Spigelian hernias are those that increase intraabdominal pressure, such as chronic obstructive pulmonary disease, obesity, pregnancy, or cirrhosis. Additional risk factors are those associated with weakened abdominal wall strength, such as female sex or age > 60 years [1]. A Spigelian hernia caused by blunt trauma is much rarer. There is limited data and evidence to support the operative technique best equipped for this repair. This case describes a 59-year-old male who presented after a fall from a tree onto a fence, which caused a traumatic Spigelian hernia. The hernia was repaired via a robotic intraperitoneal onlay mesh (IPOM) approach.

## Case report

**Figure 1:**
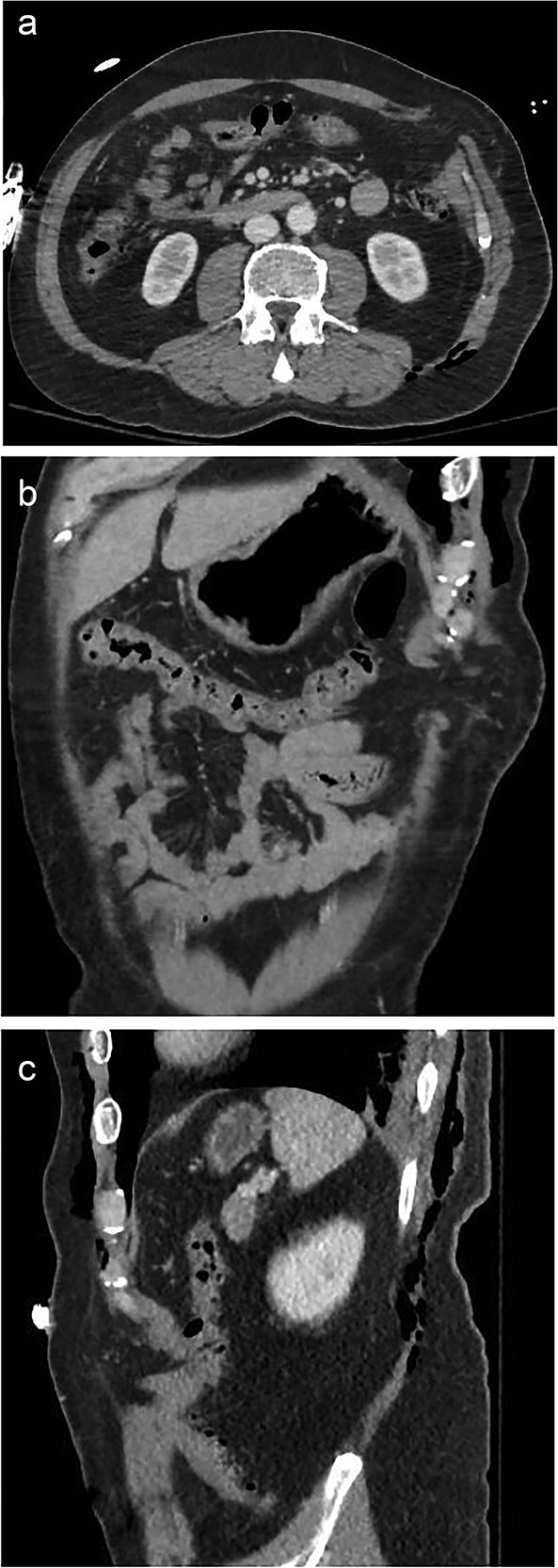
A 59-year-old male with a traumatic Spigelian hernia seen on CT. (a) Axial, (b) coronal, and (c) sagittal views of the hernia.

A 59-year-old male presented as a trauma activation after falling from a tree onto a fence. Primary and secondary surveys revealed paradoxical motion of the left chest, a large left-sided abdominal bulge, and degloving injury to the left lower extremity. Throughout the initial assessment, the patient was able to protect his own airway without evidence of significant respiratory distress and remained hemodynamically stable. Contrast-enhanced computed tomography (CT) of the chest, abdomen, and pelvis demonstrated comminuted and displaced left-sided rib fractures 4–11 consistent with flail chest and left fat-containing Spigelian hernia (Fig. 1).

He initially underwent primary closure of his lower extremity injury, followed by open reduction and internal fixation of the rib fractures. After further optimization, he was taken for repair of the Spigelian hernia during the same admission. A robot-assisted approach was chosen for repair of the Spigelian hernia.

**Figure 2:**
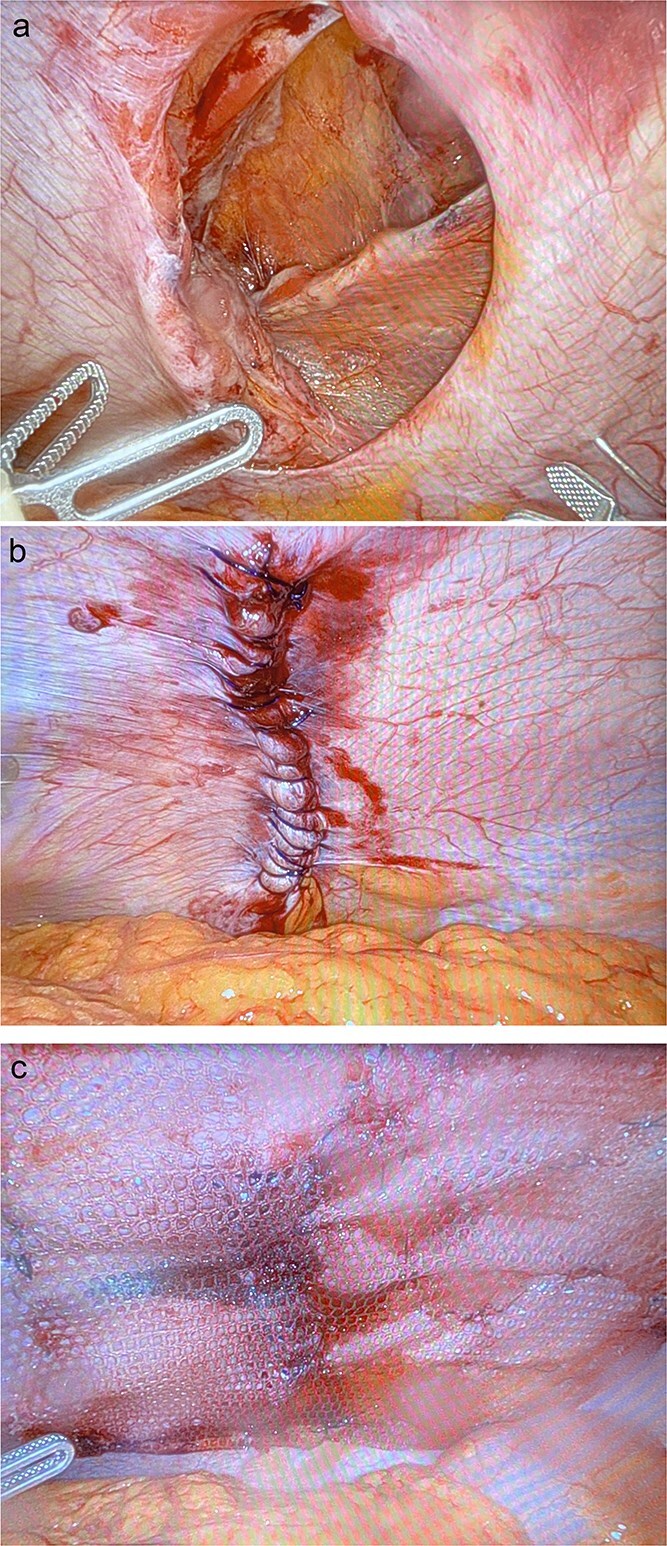
Intraoperative images of the hernia (a) prior to repair, (b) after primary closure, and (c) with onlay composite mesh placement.

**Figure 3:**
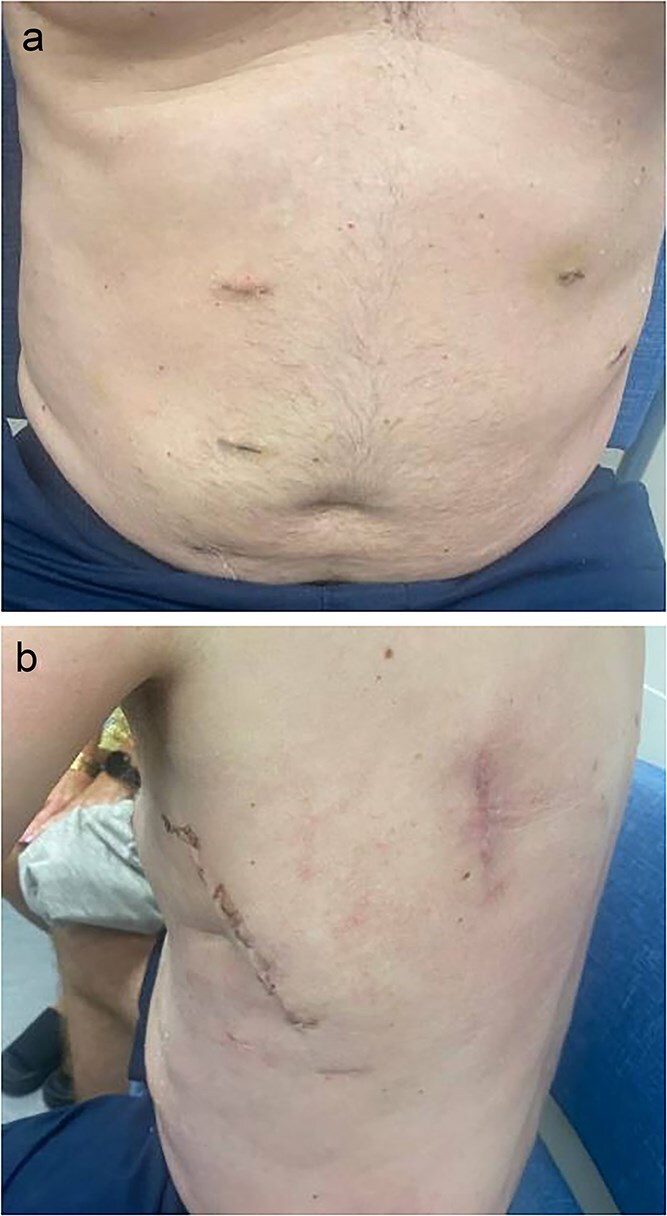
Postoperative images at 1 month follow-up with (a) frontal and (b) lateral views.

Pneumoperitoneum was established via a Veress needle at Palmer’s point. The abdomen was entered with an optical trocar. The camera port was marked 10 cm lateral to the anticipated mesh placement. There were two additional port sites placed in the right upper and right lower quadrants ~6 cm apart. The omentum spontaneously reduced from the defect that measured 8 cm in diameter (Fig. 2a). The insufflation was dropped to 10 mmHg and the defect was closed primarily with 0-Stratafix on a CT-2 needle (Fig. 2b). A 15 × 20 cm Symbotex coated mesh was prepared with a 2-0 Vicryl anchoring suture in the center. A suture passer was utilized to anchor the mesh to the abdominal wall. The mesh was secured to the abdominal wall with 3-0 V-lock suture circumferentially ensuring at least 5 cm of overlap with the hernia defect (Fig. 2c). The patient’s postoperative course was unremarkable, and he was discharged on postoperative Day 4 without issue on follow-up visits (Fig. 3).

## Discussion

There are ~15 000 reported cases of traumatic abdominal wall hernias (TAWHs) that occur every year in the USA, with the least common types being Spigelian and inguinal hernias. As with other types of hernias, factors that weaken the abdominal wall will increase the risk of Spigelian hernias. Specifically, most Spigelian hernias occur in the Spigelian belt, a zone bordered superiorly by the arcuate line, medially by the rectus abdominus, laterally by the semilunar line, and inferiorly by the interspinal plane. Within the Spigelian belt, the bordering aponeuroses are wider, which creates a natural weakness of the fibers. Injury to this area can result in traumatic Spigelian hernias, which can be classified into three different categories based on the mechanism of injury: (i) high-energy (motor vehicle accidents, fall from height), (ii) low-energy (bicycle handlebar injury), and (iii) deceleration [2]. The current case would be classified as a category one traumatic Spigelian hernia.

There are limited reported cases of traumatic Spigelian hernias described in the literature, most of which were due to blunt trauma [3]. The management of these cases, operative versus nonoperative, varied based on individual patient factors and a case-by-case basis, including concurrent injuries, complexity of the hernia, and presence of contamination [4].

The standard for management and timing of repair of TAWH in general has not yet been established. In a study of 38 patients who had TAWHs, 83% of surviving patients (30/36) underwent repair of their TAWH, and 75% (27/30) of these hernias were repaired immediately [5]. Conversely, in a different series of 34 patients with TAWH, 23.5% (8/34) underwent acute repair, and only 7.6% (2/26) managed nonoperatively went on to develop symptomatic hernias [6]. However, given that Spigelian hernias are associated with a higher risk of strangulation, a more urgent repair may be indicated [7].

There are multiple surgical approaches to repair a Spigelian hernia, such as via a midline laparotomy, direct incision over the defect, or laparoscopically [8]. In a review of 107 cases of nontraumatic Spigelian hernias, three clinical stages were identified based on hernia size (<2, 2–5, and >5 cm). Laparoscopic repair of the hernia was only suggested for stage II defects as it was neither too small (stage I) nor too large (stage III). However, no follow-up data was collected on these patients, and thus, the durability of such repairs could not be evaluated. In either open or laparoscopic techniques, use of the iliac crest as a fixation point could also be considered [9, 10]. To our knowledge, there is no study to date that compares the efficacy and durability of various repairs.

In this case, a large Spigelian hernia defect was repaired in a robotic IPOM fashion with satisfactory functional result and no evidence of recurrence at postoperative visits. While prospective comparative analyses of various techniques would be ideal, this is likely not realistic given the nature of the traumatic injury. Our case suggests the robotic IPOM technique as a safe and durable repair option.

## Conclusion

Traumatic Spigelian hernias are extremely rare, and the management is complicated by several factors, including individual patient comorbidities and concomitant traumatic injuries. Future studies are required to determine the ideal management technique with regard to long-term outcomes of traumatic Spigelian hernias. While there is no definitive consensus regarding operative management of traumatic Spigelian hernias, this report suggests that the robotic IPOM technique is safe and durable.
